# Metformin reverses the drug resistance of cisplatin in irradiated CNE-1 human nasopharyngeal carcinoma cells through PECAM-1 mediated MRPs down-regulation

**DOI:** 10.7150/ijms.48635

**Published:** 2020-09-01

**Authors:** Yingming Sun, Xiaochuan Chen, Yajuan Zhou, Sufang Qiu, Yongyang Wu, Min Xie, Guofang Zhu, Shanshan Liang, Heming Li, Dong Zhou, Zaishuang Ju, Fuguang Wang, Fang Han, Zhe Wang, Ruoyu Wang

**Affiliations:** 1 Department of Medical and Radiation Oncology, Sanming First Hospital of Fujian Medical University. Sanming 365001, China.; 2 Department of Medical Oncology, Affiliated Zhongshan Hospital of Dalian University. Dalian 116001, China.; 3 Department of Radiation Oncology, Fujian Cancer Hospital & Fujian Medical University Cancer Hospital, Fuzhou 350001, China.; 4 Department of Radiotherapy, Hubei Cancer Hospital, Tongji Medical College, Huazhong University of Science and Technology. Wuhan, 430074, China.; 5 Department of Urology Surgery, Sanming First Hospital of Fujian Medical University. Sanming 365001, China.; 6 The Key Laboratory of Biomarker High Throughput Screening and Target Translation of Breast and Gastrointestinal Tumor, Dalian University, Dalian 116001, P. R. China.; 7 Department of Medical Imaging, Affiliated Zhongshan Hospital of Dalian University. Dalian 116001, China.

**Keywords:** radiation, cisplatin resistance, metformin, PECAM-1, MRPs.

## Abstract

**Objective:** To explore a way to reverse the drug resistance for irradiated CNE-1 human nasopharyngeal carcinoma cells and try to develop a new high efficacy with low toxicity therapeutic approach. **Methods:** 300 Gy irradiated the CNE-1 human nasopharyngeal carcinoma cells, and then treated with single-agent cisplatin or metformin, or combination of both drugs. MTT assay and FCM were applied to detect cell viability and apoptosis. Western blot and RT-PCR were used to characterize the protein and mRNA expression after various drug administrations. **Results:** The results presented single-agent metformin was capable of arresting the tumor growth and inducing apoptosis in irradiated CNE-1 cells and also demonstrated a synergy effect with cisplatin. Furthermore, metformin down-regulates the PECAM-1 expression, which could regulate Multi-drug Resistance-associate Proteins (MRPs) expression leading to cisplatin resistance of irradiated CNE-1 cells. A pan-MRP inhibitor, probenecid, can resecure cisplatin resistance leading by radiation.** Conclusions:** Metformin, due to its independent effects on PECAM-1, had a unique anti-proliferative effect on irradiated CNE-1 cells. It would be a new therapeutic option to conquer cisplatin resistance for advanced NPC patients after radiotherapy.

## Introduction

Nasopharyngeal carcinoma (NPC) is one kind of the most common cancers in the south of China, and its mobility ranks first in neck and head cancers for many years 1, 2. Approximate 60% of patient diagnosis with advanced disease 3. Concurrent chemotherapy and radiotherapy, as a standard therapeutic way to flight NPS, have been recommended by various guidelines 2, 4. Although these therapeutic approaches have a good response, local control, and positive therapeutic effect 5, 6, its toxicity severely influences patients' quantity of life. Moreover, during our clinic practice, the patient undergoes a standard course of radiotherapy, would suffer a drug resistance for chemotherapy, which leads the 5-year survival rate for advanced NPC only 30% 2, 7. Our previous study noticed that radiation-induced a prominently increased level of platelet endothelial cell adhesion 1 (PECAM-1) in the CNE-1, human NPC cell line. Of note, we investigated PECAM-1 may involve in cisplatin resistance 8.

Metformin work as an "insulin sensitizer" is the most common drug for first-line therapy for T2DM patients worldwide. Recently, many scholars turn their focus on their anti-tumor effect 9-11. Epidemiologic studies indicate that people with cancer have a worse prognosis with T2DM, and in fact, metformin could decrease the risk of cancer and cancer mortality in those patients 11, 12. Furthermore, metformin presents an anti-proliferative effect on different cancer cell lines *in vitro* and *in vivo* 13-15, some mechanisms of metformin have been gradually revealed, including activating the AMPK pathway and impairing epithelial cell growth 16, 17. However, metformin as a broad spectrum anti-tumor drug, this would not be the whole story.

PECAM-1, also known as CD31, is a 130 KDa glycoprotein belonging to the immunoglobulin superfamily. Functionally, it mainly serves as an adhesion molecule, which facilitates leukocyte transmigration, angiogenesis, and integrin activation. Recently, many scholars reveal that PECAM-1 expresses in several solid tumor cells 18. Moreover, there some shreds of evidence demonstrated PECAM-1 functions not only as an essential regulator of the inflammatory response but may serves as a primary coordinator of multiple signaling pathways involved in cell metabolism and protein expression. Many clinic trails have mentioned a worn that metformin could reduce the platelets which affluent express PECAM-1 on its surface. Herein, we put forward a hypothesis, and metformin could involve in PECAM-1 regulation, which can further affect cisplatin resistance for irradiated cells 19.

However, there are seldom studies that report the impact of increased PECAM-1 expression in NPC cells, and its relevance to MDR induced by X-ray. In this article, we revealed that metformin could target PECAM-1 and reversed the resistance to cisplatin in radiation resistance NPC cells via down-regulating the MRPs family.

## Methods and Materials

### Cell culture, plasmid, and siRNA transfection

The human nasopharyngeal carcinoma CNE-1 cell line was obtained from KeyGen Biotech Co., Ltd. (Nanjing, China). Radiation resistance CNE-1 cell line (CEN-1/R) was stored by the department of oncology of Dalian University Affiliated Zhongshan hospital (Dalian, China). Both cell lines were cultured in RPMI-1640 medium (Gibco-BRL, Carlsbad, CA, USA) supplemented with 10% fetal bovine serum (FBS; Transgen, Beijing, China), 100µg/ml penicillin and 100 µg/ml streptomycin (Gibco-BRL, Carlsbad, CA, USA) at 37 ˚C in a 5% CO_2_ atmosphere.

For plasmid and siRNA transfection, lipofectamine 2000 transfect system was used. Briefly, 8×10^5^ cells were seeded into 6-well plate and cultured for 24h, then mix the lipofectamine 2000 and plasmid (5 μg) or siRNA (15 pM) with OPTI-MEM, sequentially, incubated the mixture for 30 min at room temperature, then added the mixture into the each well, complemented the OPTI-MEM up to 1 ml and cultured for 6 h. After 6 h, added PRIM 1640 with 20% FBS into each well and up to 2 ml medium, continued to culture 24 h for mRNA extraction, 48 h for protein analysis and drug administration.

The sequence for PECAM-1 siRNA: Sense cag aua cuc uag aac gga aTT; Anti-sense uuc cgu ucu aga gua ucu Gtt. Negative control siRNA was: Sense uuc ucc gaa cgu guc acg uTT; Anti-sense acg uga cac guu cgg aga Att, both siRNAs were synthesized by Invitrogen Life Technologies.

### Reagents

Metformin (cat no. D150959-5 g), Cisplatin (cat no. P4394-25 mg), Thiazolyl Blue (cat no. M2128-100 mg) and Propidium Iodide (cat no. P4170-100 mg) were purchased from Sigma-Aldrich (St. Louis, MO, USA). Probenecid (cat no. SC-202773A) was purchased from Santa Cruz, USA. The Annexin V-fluorescein isothiocyanate (FITC) Apoptosis Detection kit (cat no. KGA108) was purchased from Biovision Co., Invitrogen Life Technologies supported Lipofectmin 2000. RT-PCR Kit (cat no. K1005S) was provided by Promega Co.Ltd.

### Antibodies

The following polyclonal rabbit anti-human antibodies were used for western blot analysis: PECAM-1(cat no. AP4776a; Abcam; diluted 1:1,000), MRP-3 (cat no. SC-5776; Santa Cruz; diluted 1:500), MRP-4 (cat no. SC-20768; Santa Cruz; diluted 1:500), MRP-5(cat no. SC-376965; Santa Cruz; diluted 1:500), GAPDH (cat no. SC-365062; Santa Cruz; diluted 1:1500).

### Cell viability assay

MTT assay was used to evaluate the cell viability. The cells (5-8×10^3^) were seeded into each well of a 96-well plate and with 100 µl medium and incubated to allow the cells adhered. Then different concentrations of drugs were treated for 24 h and added15 µl MTT (5 mg/ml) then continued to incubate for 4 h at 37 ˚C. Finally, the optical density was measured using an EnSpire™ 2300 Multilabel Reader (PerkinElmer, Inc., Waltham, MA, USA) at 570 nm absorbance. Five replicates were set for each condition. The mean values were calculated, and growth curves were produced.

### Colony formation assay

CNE-1 and CNE-1/R cells (100, 200, 400, 1,000, 2,000 and 10,000 cells/well) were seeded in 6-well plates and cultured 12 h. The cells were then irradiated at 0, 1, 2, 4, 6, 8, and 10 Gray (Gy) with the Small Animal Radiation Research Platform (SARRP, 204 kV, PXI X-RAD 225Cx, CT, USA) from a photon beam. After 15 days, the colonies were fixed with 4% paraformaldehyde (PFA) for 15 mins and stained with crystal violet. The cells were photographed, and the numbers of colonies were counted. A "multitarget-single hitting" model was applied to fit the survival curve.

### Flow cytometric analysis

The CNE-1 and CNE-1/R cells were plated in 6-well plates were starved for 6 h and treated with different combinations for 24h. The cells were collected, washed twice with cold PBS, and stained with FITC-conjugated Annexin V for 20 min and propidium iodide for 15 min in the dark. The stained cells were analyzed by flow cytometry (BD FACS AriaIII, USA).

### Western blot analysis

The cells were harvested and lysed in NP40 lysis buffer, which supplemented with 1 mM phenylmethylsulfonyl fluoride (cat no. KGP610; Beyontime Biotech Co., Ltd.) and 1 mM phosphatase inhibitor cocktail (cat no. KGP602; Sangon Biotech Co., Ltd.). The harvested cells were suspended with NP-40 lysis buffer and vortex the mixture 15 s each 5 min for total 6 times, then centrifuged at 8000 rpm for 10 min and collected the supernatant. The protein concentration was qualified using a Bradford assay kit (cat no. KGPBCA; Sangon Biotech Co., Ltd.). The protein samples were mixed with loading buffer, and the proteins were separated using 8% sodium dodecyl sulfate-polyacrylamide gel electrophoresis and transferred PVDF membranes (Bio-Rad, Hercules, CA, USA). After that, the membranes were soaked in a blocking buffer (TBST with 5% fat-free milk) for 1 h. The blots were incubated at 4 ˚C overnight with the primary antibody and subsequently incubated at 37 ˚C for 1 h with the horseradish peroxidase-conjugated secondary antibody. The bands were visualized using chemiluminescence (Bio-Rad, Hercules, CA, USA), and images were captured using a ChemiDoc XRS imaging system (Bio-Rad). These were analyzed using Image Lab software (Bio-Rad).

*Reverse transcription-polymerase chain reaction (RT‑PCR).* Total RNA was abstracted by Trizol (Transgen Bio, Inc.). Total RNA (2000 ng) was reverse-transcribed into fist‑strand cDNA and taken polymerase chain reaction using an RT-PCR kit (Promega Co.Ltd) according to the manufacturer's instructions. The primers were listed in table 1. The PCR reactions were subjected to as follow: denaturation at 94˚C for 30 s, annealing (53 ˚C for MDR-1, 57 ˚C for MRP-1 and 58 ˚C for β‑actin) for 30 s and extension at 72˚C for 45 s. The PCR products were analyzed by 1.5% gel electrophoresis.

### Bioinformation analysis

The gene set of metformin physical interactions was downloaded from http://ctdbase.org/detail.go?type=chem&acc=D008687. Gene set enrichment analysis was performed on the WEB-based GEne SeT AnaLysis Toolkit. TCGA and GTEx database was used to analyze the relationship between PECAM-1 and other MRPs. Using an online tool (http://gepia.cancer-pku.cn) to realize visualization.

### Statistical analyses

Each experiment was performed in triplicates and data presented in representation of 3 individual tests. A two-tailed Student's t-test and one-way analysis of variance (ANOVA) were used to evaluate the statistical significance of different groups. Statistical analyses were performed with SPSS 16.0. P values of < 0.05 were considered as statistical significance.

## Results

### 3.1 CNE-1/R has a higher PECAM-1 expression and less sensitive for cisplatin and displayed a synergic effect with metformin

**Figure 1:**
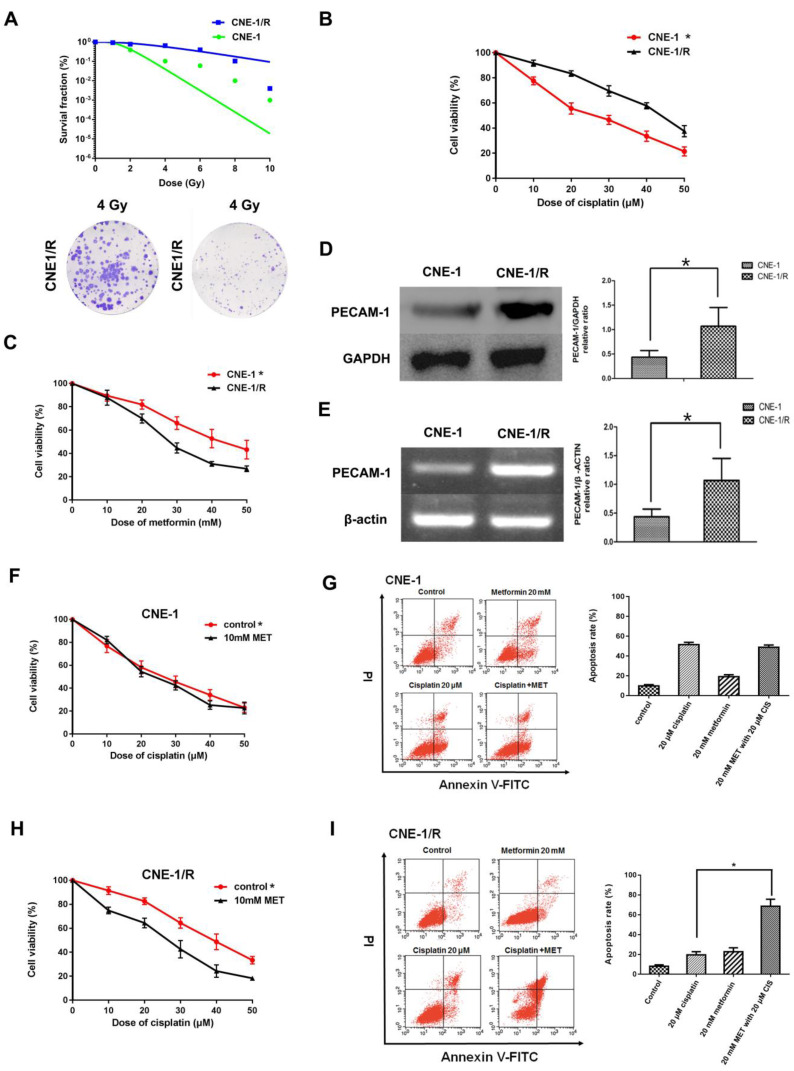
CNE-1/R has a higher PECAM-1 expression and less sensitive for cisplatin and displayed a synergical effect with metformin. (A) The multitarget-single hitting model was used to fit the survival curve. The survival fraction of CNE1 cells was significant lower than that of CNE-1/R. Representative crystal violet staining photos of CNE-1 and CNE1/R cells irradiated with 4 Gy supplemented 15 days after radiation. (B) MTT array to detect cell proliferation for CNE-1 and CNE-/R cells after 24 hours treatment of cisplatin, X-axis stood for the OD value at 570nm. *p<0.05. (C) MTT array to detect cell proliferation for CNE-1 and CNE-/R cells after 24 hours treatment of metformin, *p<0.05. (D) Western blotting analysis of protein abundance of PECAM-1, GAPDH was used as loading control, *p<0.05. (E) Reverse transcription PCR analysis of RNA abundance of PECAM-1, β-actin was used as loading control, *p<0.05. (F) MTT array to detect cell proliferation for CNE-1 cells after 24 hours administration of cisplatin and metformin. (G) FACS analysis basing on PI-Annecxin V double staining to detect the cell apoptosis of CNE-1 cells after 24 hours administration of cisplatin and metformin. Representative images of FCM was displayed. (H) MTT array to detect cell proliferation for CNE-1/R cells after 24 hours administration of cisplatin and metformin, *p<0.05. (I) FACS analyzed apoptosis of CNE-1/R cells after 24 hours administration of cisplatin and metformin. Representative images of FCM was displayed. Metformin induced more cell apoptosis combined with cisplatin, *p<0.05.

As our group had successfully established an X-ray resistance CNE-1 cell line (CNE-1/R) as shown in figure 1 A. We detected IC_50_ of cisplatin for both CNE-1 and CNE-1/R. Of note, CNE-1/R presented a cisplatin resistance phenotype with a higher IC_50_ value of 42.82 μM; as a contrast, the IC_50_ value of CNE-1 cells was only 24.2 μM (Figure 1 B). Moreover, it arose our interest that CNE-1/R cell prone to sensitive to metformin (Figure 1 C). Basing on previously gene chip result 8, we investigated the PECAM-1 expression in both protein and mRNA levels. We confirmed the result of the chip, the protein and mRNA level of PECAM-1 in CEN-1/R was significantly increased (Figure 1 D, E).

Both cell lines were incubated with different concentrations of cisplatin and 10 mM metformin. Metformin facilitated the sensitivity of the CNE-1/R cells to cisplatin (Figure 1 F); however, this synergistic effect was absent in the CNE-1 cells (Figure 1 G). The flow cytometric analysis of cell apoptosis indicated that the combination of metformin and cisplatin-induced more apoptosis than individual treatments in CNE-1/R cells (Figure 1 H). Not to our surprise, the CNE-1 cell was exempted from this combination strategy (Figure 1 I).

### 3.2 Metformin down-regulates the expression of PECAM-1 and MRPs

**Figure 2:**
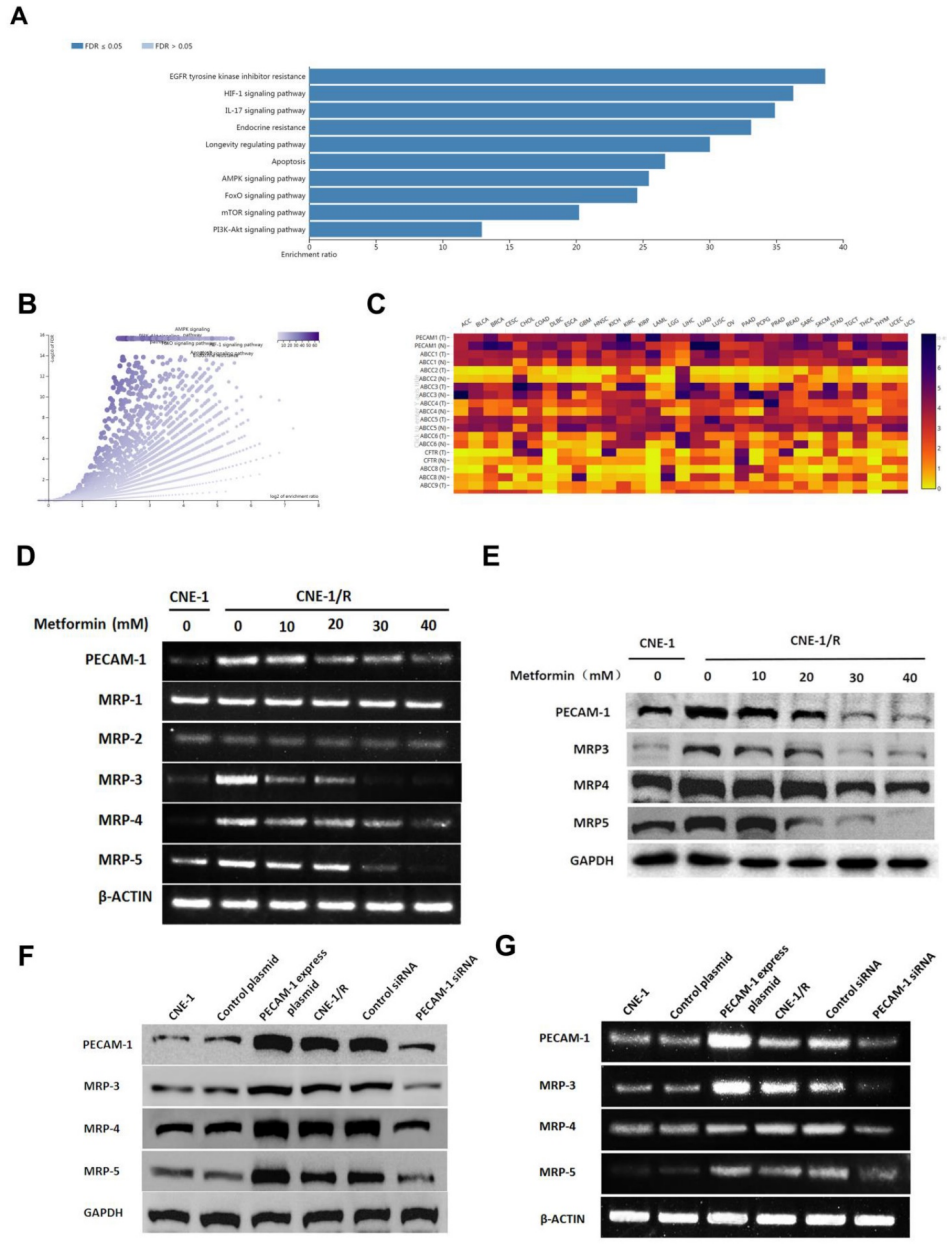
Metformin down-regulates the expression of PECAM-1 and MRPs. (A) Bar graphic of genes enrichment results of metformin regulating. (B) Vacanlo graphic of genes enrichment results of metformin regulating. (C) The heatmap of the relationship between PECAM-1 and MRPs in various normal tissues and cancers. (D) Reverse transcription PCR analysis of RNA abundance of PECAM-1 and MRPs after 24 hours disposal of various concentrations of metformin. *p<0.05. (E) Western blotting analysis of protein abundance involved in MRPs after 24 hours disposal of various concentrations of metformin. *p<0.05. (F) Reverse transcription PCR analysis of RNA abundance of PECAM-1 and MRPs in PECAM-1 knockdown or overexpression cells. *p<0.05. (G) Western blotting analysis of protein abundance of PECAM-1 and MRPs in PECAM-1 knockdown or overexpression cells. *p<0.05.

To further investigate the effect of metformin, we searched the database and listed the genes involved for enrichment analysis in table S1. We noted that metformin was involved in many kinds of drug-resistance (Figure 2 A, B). Moreover, we used the same database to analyze the relationship between PECAM-1 and MRPs. In figure 2 C, PECAM-1 presented a positive relationship with MRPs.

Cells were starved for 6 h, then treated with 10-50 mM metformin for 24 h, then harvested for further test. MRP-1, MRP-2 were failed to be regulated by metformin. However, PECAM-1, MRP-3, 4, 5 were significantly down-regulated by metformin treatment as a dose-dependent manner in both mRNA and protein levels (Figure 2 D, E).

As figure 2 F, G showed, MPR-1 and MRP-2 were independent with metformin treatment. Nevertheless, we observed that MRP-3, MRP-4, and MRP-5 were increased when we transfected the PECAM-1 express plasmids. On the other hand, we also noted the MRP-5 and MRP-4 were lessened in PECAM-1 deficiency cells in both protein and mRNA levels. We reconfirmed PECAM-1 was able to regulate MRP family in a positive manner, that suggested MRP-3, MRP-4, and MRP-5 might involve in CNE-1/R cisplatin resistance.

### 3.3 PECAM-1 affect cell apoptosis induced by single agent cisplatin and metformin plus cisplatin

**Figure 3:**
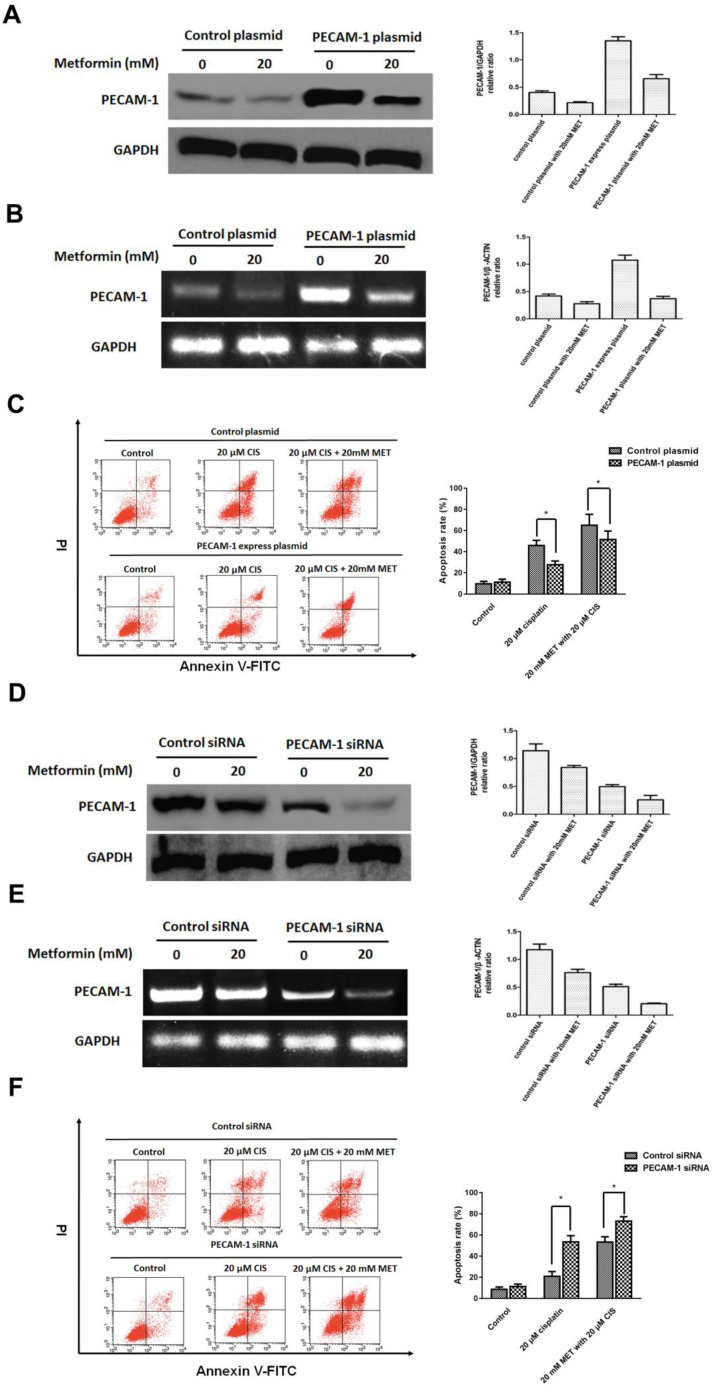
PECAM-1 affect cell apoptosis induced by single agent cisplatin and metformin plus cisplatin. (A) Western blotting was used to detect PECAM-1 expression after 20 μM metformin treatment. *p<0.05.(B) Reverse transcription PCR analysis of RNA abundance of PECAM-1 after 20 μM metformin treatment. *p<0.05. (C) FACS analysis of cell apoptosis of in CNE-1 PECAM-1 overexpressed cells after 24 hours administration of cisplatin and metformin. *p<0.05. (D) PECAM-1 siRNA was tranfected into CNE-1/R cells. Western blotting was used to detect PECAM-1 expression after 20 μM metformin treatment. *p<0.05.(E) Reverse transcription PCR analysis of RNA abundance of PECAM-1 after 20 μM metformin treatment. *p<0.05. (F) FACS analysis of cell apoptosis of CNE-1/R PECAM-1 knockdown cells after 24 hours administration of cisplatin and metformin. *p<0.05.

Herein, we established the PECAM-1 overexpression cell line. Metformin significantly decreased PECAM-1 protein and mRNA levels in overexpressed cells (Figure 3 A, B). Moreover, PECAM-1 overexpression made CNE-1 retained a cisplatin resistance, FACS results indicated that ion would reduce 18.28 ± 2.52% apoptosis rate as a negative plasmid in CNE-1 cell line. Then, we administrated the metformin and cisplatin on CNE-1^ PECAM-1 OE^ cell. As shown in Figure 3 C, the apoptosis of CNE-1 cell was almost rescued from PECAM-1 plasmid.

We also employed PECAM-1 siRNA to attenuate the PCAM-1 expression in CNE-1/R cells. As figure 3 D, E, metformin decreased the PECAM-1 in transfected cells in both protein and mRNA levels. In PECAM-1 deficiency CEN-1/R cells, cisplatin-induced more apoptosis than that of control, and the synergistic effect was further promoted (Figure 3 F).

### 3.4 Probenecid enhances apoptosis induced by single agent cisplatin and metformin plus cisplatin

**Figure 4:**
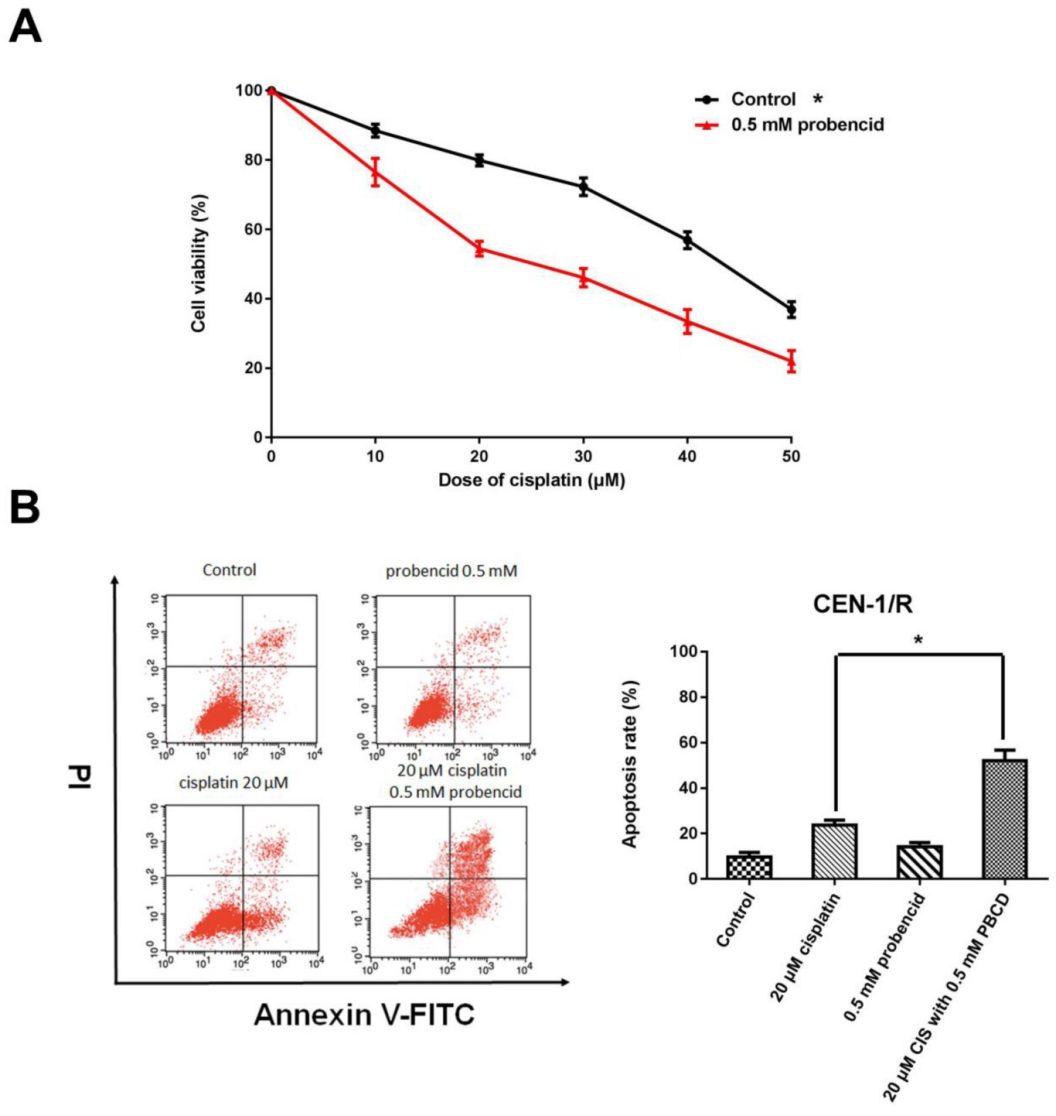
Probenecid enhances apoptosis induced by single agent cisplatin and metformin plus cisplatin. (A) MTT array to detect cell proliferation for CNE-1/R cells after 24 hours administration of 0.5 mM probenecid and cisplatin. (B) FACS analysis of cell apoptosis in CNE-1/R cells after 24 hours administration of 20μM cisplatin and 0.5 mM metformin. *p<0.05.

Probenecid was well-known as a pan-MRP family inhibitor 20. MTT result indicated that the concentration of 0.5mM influenced the cell viability (Figure 4 A). For the FACS result, single-agent probenecid exhibited a slightly toxic, but when it combines with cisplatin, it almost doubled the effect of cisplatin to CNE-1/R cell for inducing cell apoptosis (Figure 4 B, C). Inhibiting the activity of MRPs could enhance the cytotoxicity of cisplatin.

## Discussion

High energy X-rays can induce tumor cell drug resistance has been well recognized, by the fact, our previous group work have repeatedly proved that X-ray radiation can induce nasopharyngeal squamous carcinoma cells CNE-1 drug resistance 8. Tumors are a multi-gene involvement, the result of many processes of sustainable development, so people trying to employ a single tumor-associated factor to explain its particular characteristics entirely is almost impossible, so the study of drug resistance certain factors should also be considered with the multiple gene-related multidrug resistance 21. With the development of research in recent years, many scholars regard the study of radiotherapy-induced resistance to research into some of the possible mechanisms associated with resistance genes and proteins.

MDR-1, also called p-gp, is the most relevant protein to chemo-agent resistance 22. In our previous study, we reveal a limited expression of MDR1 mRNA and P-gp in untreated CNE1 cells, suggesting that the expression of MDR1 mRNA and P-gp was of minor relevance to intrinsic therapy resistance of cancer 8.

In this study, we revealed metformin could promote the cytotoxicity of cisplatin on CNE-1/R in a PECAM-1-dependent way. As we considered the primary target protein may not be PECAM-1, and then detected the MRPs after metformin treatment, metformin regulated PECAM-1, MRP-3, MRP-4, and MRP-5 in a dose-dependent mode. Moreover, CNE-1 cells obtain the cisplatin resistance after overexpressing PECAM-1; We noted that the CNE-1/R cell regained cisplatin-sensitive, PECAM-1 siRNA can enhance the combination of CNE-1/R cells cytotoxicity. It can prove CNE-1/R cisplatin resistance of PECAM-1-mediated, and metformin by down PECAM-1 and cisplatin cytotoxicity. Previous CNE-1 fails to exhibit a synergistic effect with cisplatin and metformin, might, and its related PECAM-1 expression. we also demonstrate the relationship between PECAM-1 and MRP-3, MRP-4, MRP-5. It can be concluded that the mechanism of metformin may be resistant to reverse its downward PECAM-1 mediated by decreased expression of MRPs cause sensitive to cisplatin.

Cisplatin-based cell-cycle non-specific drugs, mechanism of action essentially, when the drug into the cells, react with DNA to form two or cross-connect two chains within DNA, thereby inhibiting DNA replication and transcription, leading to DNA breakage and error code inhibit cell mitosis 23. The role of MRPs proteins are membrane pumps anticancer drugs out of tumor cells leaving the accumulated reduction of drug, which cannot achieve a sufficient concentration to inhibit tumor concentrations, thereby causing resistance 24. Our experiments performed with cisplatin resistance before and after the detection of irradiated cells, RT-PCR, Western Blot test MRPs multidrug resistance gene expression, proving once again that radiation resistance of tumor cells to make, which are associated with the early consistent with experimental results 8.

Chemotherapy is one of the primary means of treatment of various malignant tumors, which can effectively reduce the load of tumor cells in a patient, but the dose cytotoxic chemotherapy drugs often are based on the patient can withstand the MTD, because it can be most effective, the maximum the proportion of killing cancer cells, but the resulting side effects to the patient is noticeable, many patients often tolerate the pain brought about by chemotherapy, lead to treatment ineffective 25-26. Therefore, the use of a highly toxic chemotherapy sensitizer in improving efficacy while reducing the side effects of chemotherapy has become a new chemotherapeutic strategy. As it has been reported in several articles of metformin has anti-tumor function, so this study was to explore whether metformin can reverse high-energy X-rays caused by cisplatin resistance. Many scholars demonstrated *in vitro* metformin and chemotherapeutic agents can inhibit a variety of phenotypes of breast cancer cell proliferation *in vivo*, in mouse models of breast cancer in nude, metformin with a variety of chemotherapy drugs, such as carboplatin, Fama new and so when combined with the inhibition of tumor growth, prevent tumor recurrence are valid, this experiment in prostate cancer and lung cancer models are also out of the same conclusions 27. While chemotherapy drugs metformin can enhance its efficacy, but the specific mechanism has not been elucidated.

Initial chemotherapy on tumor cells typically are valid, however, the tumor cells will slowly appear to tolerate a variety of structural and functional mutually unrelated drugs 28. This multidrug resistance (multi-drug resistance, MDR) is one of the dilemmas of cancer treatment. MRPs may be considered one of the main factors of multidrug resistance 29. By flow cytometry, chemotherapy drugs have pharmacokinetics changes in those cell lines overexpressing MRPs, further, they found that compared with sensitive strains, overexpression of MRPs drugs significantly reduced the amount of intracellular accumulation of exocytosis and (or) to accelerate drug efflux 30. It is considered anti-cancer drug from the target cells are rapidly discharge the primary mechanism of MDR tumor cell production. ATP-TCA test observed MRPs protein could make cell resistant to adriamycin, epirubicin, cisplatin, actinomycin D, colchicine and other drugs. The brief mechanism of that phenomenons is mainly based on the following three aspects: First MRPs as ATP-binding protein, plays an excretory function and protection of tissues and cells, mediated efflux exogenous toxins, which acts as a carrier molecule is an energy-dependent efflux pump outward, chemotherapy drugs can discharge cells, the drug concentration inside the cell targets to reduce 31. Secondly, MRPs antineoplastic protein may enable it away from the site of action in the redistribution of the cell, resulting in a total concentration of intracellular drug has not yet decreased, but the drug cannot be combined with the target site, causing indirect resistance 32. Third, MRPs protein may also affect pH in tumor cells and cell membrane permeability, and other functions are leading to drug resistance 33.

This study was to ascertain the effect of metformin PECAM-1 reverse the CNE-1/R cells resistant to cisplatin and proved PECAM-1 resulted in increased expression of MRPs family of high-energy X-rays to find the cause of cisplatin-resistance protein. For the future of animal experiments and clinical applications, provide a theoretical basis. Taking into account the PECAM-1 versatility, and an essential role in tumor development, continue to explore specific to PECAM-1 drugs, and developing for its polyclonal antibody may provide more individualized treatment options for cancer and to further improve the prognosis of patients with malignant tumors.

## Supplementary Material

Supplementary table S1.

## Figures and Tables

**Table 1:** List of primers

Gene symbol	Forward strand	Reverse strand
PECAM-1	AAGTGGAGTCCAGCCGCATATC	ATGGAGCAGGACAGGTTCAGTC
MRP-1	CCGTGTACTCCAACGCTGACAT	ATGCTGTGCGTGACCAAGATCC
MRP-2	GCCAACTTGTGGCTGTGATAGG	ATCCAGGACTGCTGTGGGACAT
MRP-3	GAGGAGAAAGCAGCCATTGGCA	TCCAATGGCAGCCGCACTTTGA
MRP-4	CTGTTGGAGGATGGTGATCTGAC	CTGCTAACTTCCGCATCTACTGC
MRP-5	GGCTGTATTACGGAAAGAGGCAC	TCTTCTGTGAACCACTGGTTTCC
β-actin	CACCATTGGCAATGAGCGGTTC	AGGTCTTTGCGGATGTCCACGT
GAPDH	GTCTCCTCTGACTTCAACAGCG	ACCACCCTGTTGCTGTAGCCAA
