# Supplementary material for: Metformin reverses the drug resistance of cisplatin in irradiated CNE-1 human nasopharyngeal carcinoma cells through PECAM-1 mediated MRPs down-regulation
Source: Int J Med Sci. 2020 Sep 1;17(16):2416–26. doi: 10.7150/ijms.48635 (PMC7532475; doi:10.7150/ijms.48635)
Supplement: Supplementary file 1 — Supplementary table S1. [file ijmsv17p2416s1.pdf]

Symbol	Name
ABCB9	ATP-binding cassette, sub-family B (MDR/TAP), member 9
ABCC3	ATP-binding cassette, sub-family C (CFTR/MRP), member 3
AGRN	agrin
AK1	adenylate kinase 1
AK2	adenylate kinase 2
AKR1B10	aldo-keto reductase family 1, member B10 (aldose reductase)
AP1G2	adaptor-related protein complex 1, gamma 2 subunit
AP1S1	adaptor-related protein complex 1, sigma 1 subunit
APBB3	amyloid beta (A4) precursor protein-binding, family B, member 3
APOE	apolipoprotein E
ARG2	arginase 2
ARHGAP10	Rho GTPase activating protein 10
ARNT	aryl hydrocarbon receptor nuclear translocator
BAX	BCL2-associated X protein
BLVRA	biliverdin reductase A
BRD4	bromodomain containing 4
C11ORF30	chromosome 11 open reading frame 30
C18ORF25	chromosome 18 open reading frame 25
C22ORF29	chromosome 22 open reading frame 29
CASP8	caspase 8, apoptosis-related cysteine peptidase
CCL20	chemokine (C-C motif) ligand 20
CCNJL	cyclin J-like
CXCR4	chemokine (C-X-C motif) receptor 4
CYP2E1	cytochrome P450, family 2, subfamily E, polypeptide 1
DAZAP2	DAZ associated protein 2
DDX11	DEAD/H (Asp-Glu-Ala-Asp/His) box helicase 11
DNMT3A	DNA (cytosine-5-)-methyltransferase 3 alpha
EML3	echinoderm microtubule associated protein like 3
ETV1	ets variant 1
EVPL	envoplakin
EYA4	EYA transcriptional coactivator and phosphatase 4
FAM188A	family with sequence similarity 188, member A
GK	glycerol kinase
GOSR2	golgi SNAP receptor complex member 2
GPD2	glycerol-3-phosphate dehydrogenase 2 (mitochondrial)
GUSBP3	glucuronidase, beta pseudogene 3
HIRA	histone cell cycle regulator
HNMT	histamine N-methyltransferase
IFI27	interferon, alpha-inducible protein 27
IFI44L	interferon-induced protein 44-like
IFI6	interferon, alpha-inducible protein 6
IFITM1	interferon induced transmembrane protein 1
ING4	inhibitor of growth family, member 4
ITSN1	intersectin 1 (SH3 domain protein)
KIAA1462	KIAA1462
KIDINS220	kinase D-interacting substrate, 220kDa
KLF3	Kruppel-like factor 3 (basic)

KLHL25	kelch-like family member 25
KLK10	kallikrein-related peptidase 10
LMNB1	lamin B1
LPIN2	lipin 2
LTBP4	latent transforming growth factor beta binding protein 4
MATR3	matrin 3
METTL3	methyltransferase like 3
MID2	midline 2
MMP13	matrix metallopeptidase 13
MTAP	methylthioadenosine phosphorylase
MYL12A	myosin, light chain 12A, regulatory, non-sarcomeric
MYL4	myosin, light chain 4, alkali; atrial, embryonic
NPAS2	neuronal PAS domain protein 2
NR2C1	nuclear receptor subfamily 2, group C, member 1
NT5M	5',3'-nucleotidase, mitochondrial
NUBPL	nucleotide binding protein-like
NUS1P3	nuclear undecaprenyl pyrophosphate synthase 1 homolog (S. cerevisiae) pseudogene 3
OAS1	2'-5'-oligoadenylate synthetase 1, 40/46kDa
PASK	PAS domain containing serine/threonine kinase
PDCD4	programmed cell death 4 (neoplastic transformation inhibitor)
PDLIM7	PDZ and LIM domain 7 (enigma)
PFDN6	prefoldin subunit 6
PHF21A	PHD finger protein 21A
PIP5K1A	phosphatidylinositol-4-phosphate 5-kinase, type I, alpha
PLEKHO1	pleckstrin homology domain containing, family O member 1
PPCS	phosphopantothenoylcysteine synthetase
PPP3R1	protein phosphatase 3, regulatory subunit B, alpha
PRF1	perforin 1 (pore forming protein)
PRKCH	protein kinase C, eta
PRR15L	proline rich 15-like
PRR7	proline rich 7 (synaptic)
PSD3	pleckstrin and Sec7 domain containing 3
PTP4A3	protein tyrosine phosphatase type IVA, member 3
SLC35A2	solute carrier family 35 (UDP-galactose transporter), member A2
SLC39A7	solute carrier family 39 (zinc transporter), member 7
SLC48A1	solute carrier family 48 (heme transporter), member 1
SRRM2	serine/arginine repetitive matrix 2
SULT1A2	sulfotransferase family, cytosolic, 1A, phenol-preferring, member 2
TCAF1	TRPM8 channel-associated factor 1
TMEM254	transmembrane protein 254
TNFRSF25	tumor necrosis factor receptor superfamily, member 25
TNKS2	tankyrase, TRF1-interacting ankyrin-related ADP-ribose polymerase 2
TRIM44	tripartite motif containing 44
TTC38	tetratricopeptide repeat domain 38
UBAP1	ubiquitin associated protein 1
UBOX5	U-box domain containing 5
ZNF107	zinc finger protein 107
ZNF358	zinc finger protein 358

ZNF592	zinc finger protein 592
ZNF654	zinc finger protein 654
ZNF671	zinc finger protein 671
